# The Chinese version of the autonomy preference index for advanced cancer patients: a study on cultural adaptation based on cognitive interview

**DOI:** 10.1186/s40359-025-02391-y

**Published:** 2025-04-02

**Authors:** Chao Yan, Yonghong Li, Ji Ai, Shenghuan Yang

**Affiliations:** 1 Nursing Department, Guizhou Aerospace Hospital, Guizhou Zunyi, 563000 China; 2 Nursing Department, Affiliated Hospital of Zunyi Medical University, Guizhou Zunyi, Guizhou 563000 China

**Keywords:** Hospice care, Advanced cancer, Autonomy preference, Cognitive interview, Qualitative research

## Abstract

**Background:**

The global cancer burden is becoming increasingly severe. In the context of patient-centred medicine, respecting patients’ autonomy and preferences is of paramount importance. However, there is currently a lack of scientific tools in China to measure the autonomous preferences of advanced cancer patients. We aim to optimise assessment tools for patients’ autonomous preferences and validate their effectiveness, thereby filling a gap in related research, in hopes of improving the quality of medical care in China.

**Objectives:**

① To assess the semantic clarity of entries of the Chinese Autonomy Preference Index (API) and determine whether patients can accurately comprehend their content. ② To validate the application effect of cognitive interviews in the translation of the scale into the Chinese culture and context.

**Methods:**

In March and April 2023, we selected 17 advanced cancer patients by convenience sampling in Zunyi, Guizhou, China, to participate in this study. We assessed their understanding of each item in the Chinese API scale through cognitive interviews and made the corresponding revisions to the scale items based on the interview results.

**Results:**

The respondents’ understanding of various API entries after translation and adaptation was assessed. Based on the interview results, ambiguous entries were revised to create a refined Chinese version of the API. Ultimately, the API comprises two dimensions and 23 entries. The results of the first round of interviews revealed doubts or ambiguities in the semantic expression and understanding of 5 items, which were then revised following discussions by the research team. The second round of interviews confirmed that the interviewees could correctly understand the content of the entries without further modifications.

**Conclusions:**

① Cognitive interviews can address discrepancies in the understanding of scale items among the target population and mitigate measurement errors stemming from item content ambiguity. ② Targeted questionnaire revisions have improved the accuracy, reliability, and applicability of the Chinese version of the API questionnaire. The Chinese version of the Autonomy-Preference-Index offers clinical healthcare professionals an effective measurement tool to assess the autonomous preferences of advanced cancer patients.

**Supplementary Information:**

The online version contains supplementary material available at 10.1186/s40359-025-02391-y.

## What is already known

• The Autonomy Preference Index (API) has been extensively utilised in various populations outside of China, such as primary care patients and mental illness patients.
• Respecting patient autonomy preferences is a vital component in enhancing the quality of medical care.
• In a patient-centred medical environment, nurses play a crucial role in safeguarding patients’ autonomous preferences.

**Supplementary Information:**

The online version contains supplementary material available at 10.1186/s40359-025-02391-y.

## What this paper adds

• Introduces the API to China, offering Chinese medical staff a practical tool to measure the autonomous preferences of advanced cancer patients.
• Adapts the API to China’s country-specific characteristics and cultural nuances through cognitive interviews, ensuring its relevance and applicability in the Chinese context.
• Marks the first application of the API to advanced cancer patients in China, paving the way for a better understanding and greater respect of their autonomous preferences in medical care.

## Introduction

Cancer is the primary cause of mortality worldwide, impeding progress in extending life expectancy [1, 2]. According to Global Cancer Statistics 2020, the number of newly diagnosed cancer cases increased to 19.3 million in 2020, leading to almost 10 million fatalities [3]. In the same year, the age-standardised incidence rate in China was 204.8 per 100,000, placing it 65th globally, whereas the age-standardised mortality rate was 129.4 per 100,000, ranking China 13th [4]. Despite advanced cancer patients opting for treatments such as chemotherapy and targeted therapies [5, 6], a significant proportion of them endure physical, psychological, social, and spiritual distress [7, 8]. Consequently, the quality of life for patients with advanced cancer remains low.

As the disease progresses, advanced cancer patients place significant importance on both the quality of their personal life and the quality of their impending death [9, 10]. Research has demonstrated that these patients hold high expectations for a peaceful and dignified end-of-life experience [11]. Respecting their autonomous preferences is pivotal in enhancing the overall quality of healthcare they receive [12]. The quality of medical care provided to these patients can directly influence the quality of their death, which serves as an indicator of whether the expectations and needs of terminal cancer patients are being met, essentially reflecting their quality of life during the final stage.

Given their disease status, advanced cancer patients exhibit varying preferences regarding the acquisition of disease information and their involvement in medical decision-making processes [13]. In light of this, determining how to influence patients’ participation in decision-making and their access to medical information while also considering medical efficacy and safeguarding patients’ autonomous preferences has become a prominent socioethical issue [14]. Consequently, the urgent scientific challenge lies in developing a method to accurately measure patients’ autonomous preferences.

Any decision concerning medical care should take into account patients’ autonomous preferences regarding their involvement in the decision-making process. However, in China, there is a scarcity of published research results on scientifically measuring the degree of autonomous preference among cancer patients. This lack of research often leads to patients not receiving comprehensive medical and nursing information [15]. Limited information sharing among doctors, nurses, and patients can exacerbate doubts about the authenticity of shared information, thereby complicating the doctor-patient relationship. Cancer patients have diverse expectations regarding medical care decisions and the acquisition of related information. A personalised and scientifically predetermined medical care plan can alleviate anxiety and depression among patients to a certain extent and enhance medical cooperation and compliance [16].

Cognitive interviewing is a qualitative interview method with a psychological orientation that focuses on the cognitive processes of respondents when answering survey questions. This technique typically involves face-to-face interviews, allowing researchers to observe the entire thought process and response behaviour of respondents and identify potential cognitive biases. By assessing the target population’s understanding of questionnaire items, the questionnaire can then be revised and adjusted to uncover additional potential issues, thereby reducing sources of response error and enhancing the reliability of the results. This approach also improves the problem detection rate and the quality of questionnaire design.

In 1989, American scholar J. Ende developed the Autonomous Preference Index (API) [17], which measures patients’ preferences in two dimensions: medical decision-making and information acquisition level. The API is widely used internationally [18], but there is limited research on measurement tools related to patient autonomy preference in China. The scale has been adopted and utilised in Germany [19], France [20], Japan [21], and other countries [22] and has been effectively validated in clinical practice for various populations, including primary care patients [23], mental health patients [24], and advanced cancer patients [25]. Specifically, Isabelle Colombet in France applied this scale to advanced cancer patients and reported that it has good reliability and validity [20]; however, a Chinese version of the API has not yet been developed. While there are global tools to measure decision-making or information-seeking levels in advanced cancer patients, there are few tools to measure the decision-making and information preferences of this target group.

Therefore, this study employs cognitive interviews in the cultural adaptation process of the API to enhance the understanding and cultural adaptability of the Chinese version of the API within the Chinese context. The objective is to evaluate the application effect of this method in sinicising the scale. The following report details the study.

## Study subjects and methodology

### Setting

This study is grounded in the cultural adaptation framework of cognitive interview research and aims to ascertain the presence of semantic ambiguity in the Chinese version of the Autonomous Preference Index for advanced cancer patients. The study was conducted at a tertiary hospital located in Guizhou, China from March to April 2023. The participants were sourced from the hospice unit, chemotherapy unit, and oncology clinic of the hospital, which serves as a prominent tertiary cancer centre in Guizhou Province that attracts patients from across the country.

### Measurement Instrument and Procedure

Before the cognitive interviews, the scale underwent a rigorous translation and adaptation process adhering to the Brislin translation model’s bidirectional translation method [26]. The translation and adaptation procedure encompassed the following steps:

① Literal Translation: The scale was initially translated into Chinese by two bilingual researchers, both of whom hold master’s degrees in nursing, with excellent English proficiency.

② Debugging: A nursing expert then compared the two literal translations, ensuring accuracy and addressing any discrepancies through iterative refinements.

③ Back Translation: Subsequently, two faculty members from the School of Foreign Languages translated the Chinese version back into English, ensuring that the original meanings were preserved.

④ Refining Debugging: A nursing graduate who was unfamiliar with the scale integrated and adjusted the translated version to enhance its cultural relevance and readability.

⑤ Delphi Expert Consultation: The articulation, cultural equivalence, and relevance of the scale items were meticulously evaluated through Delphi expert consultation. The background information of the Delphi experts is presented in Table 1.

⑥ Focus Group Discussion: A focus group discussion was conducted to deliberate on the outcomes of the Delphi expert consultation and make necessary adjustments to the content of the scale items.

Ultimately, the sinicised scale comprises two components: the 8-item Information-Seeking Preference Scale (IS) and the 15-item Decision-Making Scale (DM), with a total of 23 items. These items collectively aim to comprehensively assess the preferences of advanced cancer patients in terms of information seeking and decision-making.

**Table 1:** Background information of experts

Item	Classification	Classification	Constituent ratio
Working life	10–19 years	6	30%
	20–29 years	5	25%
	≥ 30 years	9	45%
Research field	Medical oncology	4	20%
	Oncologic nursing	5	25%
	Hospice care	7	35%
	Sociology and Psychology	4	20%
Age	30–39 years	3	15%
	40–49 years	7	35%
	≥ 50 years	10	50%
Degree of education	bachelor degree	9	45%
	master’s degree	4	20%
	doctoral degree	7	35%
Degree of education	Associate senior title	7	35%
	high professional title	13	65%

### Sampling and recruitment

According to the guidelines for the translation and adaptation of cross-cultural research tools, cognitive interviews should be conducted with a target group ranging from 10 to 40 participants during the language and cultural adaptation process [27]. In this study, a total of 17 patients with advanced cancer were recruited, with 10 participants in the first round and 7 in the second round. Convenience sampling was employed in both rounds of face-to-face interviews to ensure the sample’s diversity and representativeness.

The recruitment procedure is outlined below:

① Convenience Sampling: To ensure the representativeness of the sample, patients with advanced cancer who voluntarily agreed to participate in the study were recruited through convenience sampling. Informed consent was obtained from each participant before their involvement.

② Explanation of the Research Purpose and Procedures: The interviewer provided a detailed explanation of the research purpose and interview procedures and assured participants that all the information would be kept strictly confidential and used solely for scientific research purposes.

③ Completion of the Questionnaire: Participants were informed about all the questions in the questionnaire. They were instructed to mark any items that they did not understand. After completing the questionnaire, the interviewer conducted a structured interview according to the interview outline, with the interview duration controlled to be within 30 min.

④ Data Saturation Principle: Information collection for each round of interviews was guided by the principle of data saturation. After each interview, the data were transcribed, and any issues identified were addressed. The subsequent interviews continued until the participants could clearly understand the questionnaire instructions and all the items.

Inclusion Criteria: Patients diagnosed with advanced cancer by pathology (defined as having multiple systemic metastases and incurable cancer). Patients must be at least 18 years old and have good cognitive function (i.e., have a Montreal Cognitive Assessment score greater than 26). They must have Chinese as their mother tongue, and have good language expression ability, and have completed at least a primary school level of education. Patients must provide informed consent to voluntary participate in the study.

Exclusion criteria: Patients with advanced cancer with a history of mental illness were excluded from the study.

### Data Collection

In this study, data were collected through semi-structured, one-to-one interviews conducted during patients’ hospitalisation. Sample selection was based on clear inclusion and exclusion criteria to recruit cancer patients who volunteered to participate. The sample size was determined by code saturation, which occurred when no new issues emerged and the codebook began to stabilise. After analysing the interview results, the research group agreed that code saturation was achieved after 10 advanced cancer patients were recruited in the first round and an additional 7 patients were recruited in the second round. During the second round of cognitive interviews, the respondents demonstrated a better understanding of the scale items’ contents when they were prompted about ambiguities. Consequently, the research team decided to terminate the data collection.

The interviews were conducted using interpretation, probing techniques, and interviewee reporting. They took place in a quiet, private office and were recorded for later transcription and analysis. The face-to-face, semi-structured interview method allowed for flexibility in exploring the participants’ perspectives. Two interviewers, who were both nursing researchers with comprehensive research training and extensive experience in facilitating qualitative research interviews, conducted the two rounds of cognitive interviews.

Based on a literature review and API entries, the research team developed an interview outline that included the following:

General exploration questions about the overall API framework design (e.g., scale length, font).

Observational questions were used to understand why the participants hesitated in their choices.

Probing questions were used to assess participants’ understanding of specific items’ content.

Exploratory questions about participants’ thoughts and details when answering specific items.

Comfort probing questions were asked to identify any discomfort during the interview process.

Content exploration questions were used to assess the relevance of API entries to decision-making and information acquisition and gather suggestions for adding or removing items from the scale.

### Data Analysis

After the cognitive interviews, data analysis was conducted in three steps:

Transcription: The researcher listened to the interview recordings multiple times and transcribed them within 24 h. Coding: The transcribed data were sorted and coded using a problem evaluation system. Issues identified in the interview results were categorised into eight areas: reading, instructions, clarity, hypothesis/logic, knowledge/memory, sensitivity/bias, response category, and others.

Revision: The research team verified the existing problems with the interviewees, conducted group discussions, and made decisions to finalise the revision plan for relevant items.

### Ethical considerations

All participants signed informed consent forms approved by the Medical Ethics Committee (KLLY-2022-201). Written informed consent was obtained before the interviews. The participants were informed that they could refuse to answer any questions or withdraw from the study at any time. To ensure confidentiality, the interview transcripts were de-identified, and only the research team members had access to the data.

## Results

### Basic information of the respondents

Between March and April 2023, a total of 17 cancer patients were recruited as respondents from a tertiary hospital located in Zunyi, Guizhou Province. Each respondent was interviewed cognitively in separate rounds, ensuring that no two rounds coincided with the same participants. In the first round, 10 respondents (labelled I-P1 to I-P10) participated, with an average age of 49.7 years. The age range within this group was 2274 years. In the second round, 7 respondents (labelled II-P1 to II-P7) participated, with an average age of 56.86 years. The oldest participant in this group was 70 years old, whereas the youngest was 29. Each patient was interviewed for at least 15 min. The general information of all the respondents is as follows: presented in Table 2.

**Table 2:** General information on respondents in 2rounds(case: *N* = 17)

Participant	Gender	Age	Education	Diagnosis	Monthly income($)
I-P1	Female	46	Junior middle school	Breast cancer	414–691
I-P2	Female	25	Undergraduate course	Breast cancer	414–691
I-P3	Male	59	Primary school	Esophagus cancer	414–691
I-P4	Female	74	Senior middle school	Lung cancer	691–1105
I-P5	Female	54	Junior middle school	Breast cancer	691–1105
I-P6	Male	73	Primary school	Lung cancer	<414
I-P7	Male	66	Senior middle school	Skin cancer	>1381
I-P8	Male	22	Undergraduate course	Nasopharyngeal cancer	691–1105
I-P9	Female	42	Senior middle school	Glioma	691–1105
I-P10	Female	36	Senior middle school	Cervical cancer	691–1105
II-P1	Male	59	Junior middle school	Cancer of mouth	691–1105
II-P2	Female	29	Senior middle school	Nasopharyngeal cancer	691–1105
II-P3	Female	62	Primary school	Cervical cancer	691–1105
II-P4	Male	70	Primary school	Rectal cancer	691–1105
II-P5	Female	68	Primary school	Ovarian cancer	691–1105
II-P6	Male	64	Primary school	Lung cancer	<414
II-P7	Female	46	Junior middle school	Breast cancer	691–1105

### Interview results

#### Results from the first round of interviews

A total of 17 patients with advanced cancer were recruited for the study. Most of the participants had no objections to the API framework design, the number of items, the fonts used, or other related aspects. However, some participants required assistance in understanding the content, language expression, and professional vocabulary of one of the articles. Based on the findings from the first round of interviews, the research team reviewed and discussed the recorded content. The team subsequently modified the content of ambiguous entries, as detailed in Table 3.

**Table 3:** Autonomous preference index entries doubt and revision plan

Original entry	Revised instructions and solutions	Revised entry
5. If you get sick, you want your doctor to have more decision-making authority as your condition worsens.	In response to the understanding that “doctors can get more decision-making management power”, seven respondents said that “doctors get more decision-making management power” means that personal rights are entrusted to doctors. After analyzing the results of interviews, group discussions, and expert consultation, the project team decided to incorporate the opinions. After the amendment changed it to “make more medical decisions for you”.	If you get sick, you want your doctor to make more medical decisions for you as your condition worsens.
12. If you have cancer-related fatigue, who decides whether you should receive symptomatic treatment?	In response to the understanding of “cancer-related fatigue”, eight respondents said: “cancer-related fatigue is not fatigue”, and some respondents proposed that the vocabulary was too professional and difficult to understand. After group discussion, this opinion was incorporated, and a supplementary explanation of cancer-related fatigue was added after the article’s content.	If you have cancer-related fatigue (sleep disturbances, low mood, memory loss, etc.), who decides whether you should receive symptomatic treatment?
13. Who decides whether or not you take first aid?	As for “first aid measures”, five respondents said that in addition to first aid measures, some life support treatment was included in the terminal stage. Three respondents asked, what are the first aid measures? After group discussion, the opinions were incorporated, and the contents of the articles and supplementary explanations were added.	Who decides whether you should take first aid measures or life support treatment? (e.g. tracheal intubation, cardiopulmonary resuscitation, hemodialysis, etc.)
15. Who develops and determines your advance medical directive?	Regarding “advance medical Directives”, eight respondents said they had heard of advance medical directives. An advance medical directive is a pre-set medical care plan that has been used for a long time in foreign countries for advanced cancer. This research is in the initial stage in China, and the public awareness rate needs to be higher. After the group discussion, it was decided that the advanced medical Directive will be revised and supplemented due to the relatively mature research progress on living wills and advanced medical care plans in China.	Who makes your default medical care plan? (Living Will, advance medical Directive, advance medical care plan, etc.)
17. You should be fully aware of the effects of your illness on the inside of your body.	For “inside the body,” four respondents said the cancer was more than physical. After a group discussion, the opinion was incorporated, and the word “inside the body” was replaced with “mind and body”.	You should be fully aware of the impact your illness has on your body and mind.

## Discussion

### Cognitive interviews can effectively resolve understanding errors and help patients understand the content of the items

During the translation and adaptation process, cognitive interviews were conducted to assess the understanding of the content of each item in the Chinese API. In the first round of the interviews, based on the feedback from the respondents, we modified the contents of five items, including ambiguous expressions of the original scale items and supplementary interpretations of the professional vocabulary of the original scale items. The respondents indicated that they could understand the scale and complete it within 5 min. These findings suggest that cognitive interviews can effectively enhance late-stage cancer patients’ understanding of the content of the API, thereby improving the efficiency and clinical value of the API.

During the interviews, the interviewer identified five common issues that caused respondents to hesitate or provide incorrect answers. There are two potential reasons for these issues. First, the design of the item may have led to misunderstanding among the interviewees, necessitating modifications based on their feedback and group discussion. Second, a small number of respondents with lower education levels may need assistance in understanding certain words [28]. In such cases, modifications to the items may not be necessary at this stage, and appropriate explanations or supplementary information should be provided as needed. Therefore, modifications to the items should be selective and based on the specific situation.

This scale includes four reverse-scored items, and respondents needed clarification on some of these items, leading to misunderstandings. This indicates a difference in understanding between the scale designers and the respondents. Therefore, it is crucial to compile and modify items from the perspective of the target population to ensure that they are clear and easy to understand.

### Cognitive interviews can further improve the application of the Chinese API in advanced cancer patients

In 1989, American scholar J. Ende and colleagues developed the Autonomy Preference Index to measure patients’ desire to make medical decisions and access medical information [18], reflecting their autonomous preferences. Initially, the API was widely used in primary care settings. However, with the increasing incidence of cancer worldwide, the cancer burden has become increasingly significant. Ensuring that cancer patients can make medical decisions based on their values has become increasingly important [29].

This study is the first in China to focus on advanced cancer patients using the API. Although some respondents had doubts about specific terms such as “decision-making management right” and “life-sustaining treatment,” most were willing to accept these items after the researchers provided additional explanations of the terminology. Furthermore, the respondents were even eager to discuss topics such as “the meaning of death,” “how to improve the quality of their end-of-life care,” and “how to complete a preset medical care plan” with the researchers.

These findings suggest that cognitive interviews can further enhance the cultural adaptability of the Chinese version of the API for advanced cancer patients. Moreover, they highlight the importance of establishing a scientific and healthy view of death, which can help people face and accept death more rationally.

### The Chinese version of the autonomy-preference-index has good practicability and operability

Cancer poses a significant threat to the health of the Chinese population and is a major public health concern. As key stakeholders in medical services, cancer patients must actively engage in decision-making about their treatment and information acquisition, collaborating with medical staff to make informed medical choices [30–31]. However, research on cancer patients’ autonomous preferences in China is still in its early stages, and there is a relative lack of research on measurement tools for these preferences.

To address this gap, a rigorous and scientific translation and introduction process was followed for the Autonomy Preference Index. The translation team, which was proficient in both Chinese and English, translated the index through a series of steps, including translation, debugging, back-translation, further debugging, review by the original author, the Delphi method, cognitive interviews, a pre-survey, and reliability and validity testing. The Delphi expert consulting team was composed of highly motivated experts from diverse research fields, including clinical oncology, cancer nursing, hospice care, sociology, and psychology, with strong expertise and a broad geographical representation from Shaanxi, Anhui, Zhejiang, Sichuan, Guangxi, and Guizhou Provinces.

The application of the Delphi method involved two rounds of expert consultation, during which 20 experts participated. Among them, 65% held senior professional titles, and 55% possessed master’s degrees or higher. Their expertise ensures the robustness and reliability of the consultation process.

Furthermore, the cognitive interview process was conducted rigorously and efficiently. A total of 17 cancer patients participated in two rounds of cognitive interviews, which aimed to ensure the practicability and operability of the Chinese version of the API in subsequent clinical use among patients.

In summary, this study demonstrates a comprehensive and scientific approach to adapting and validating the API for use in the Chinese context, with a focus on cancer patients. The rigorous translation and introduction process, combined with expert consultation and cognitive interviews, provides a strong foundation for future research and clinical application of the Chinese version of the API.

## Limitations

When recruiting participants, to ensure the sample’s diversity, the research team tried to consider multiple factors such as gender, age, disease, education and monthly income. However, since this study only used convenience sampling in one hospital, the results of this study have some limitations, and they need to be evaluated in more samples to achieve the utility of the tool and improve patients’ understanding of the tool.Subsequently, we will continue to improve the scale through pre-investigation, reliability and validity tests.

## Conclusions

Cognitive interviews can address the differences in understanding of scale items among the target population and reduce measurement errors caused by ambiguity in the content of the items. The Chinese version of the Autonomy-Preference-Index provides an effective measurement tool for clinical healthcare professionals to measure the autonomous preferences of advanced cancer patients.

## Electronic supplementary material

Below is the link to the electronic supplementary material.

Supplementary Material 1

## Data Availability

The datasets generated and analysed during the current study are not publicly available due but are available from the corresponding author on reasonable request.

## Abbreviations

API: Autonomy preference-Index
IS: Information seeking preference scale
DM: Decision making scale
