# Supplementary material for: The Chinese version of the autonomy preference index for advanced cancer patients: a study on cultural adaptation based on cognitive interview
Source: BMC Psychol. 2025 Apr 2;13:322. doi: 10.1186/s40359-025-02391-y (PMC11967037; doi:10.1186/s40359-025-02391-y)
Supplement: Supplementary file 1 — Supplementary Material 1 [file 40359_2025_2391_MOESM1_ESM.docx]

**Expert Consultation Form**

Dear experts，

shalom! Thank you for taking time out of your busy schedule to read this consultation form!

I am Yan Chao, a graduate student from the School of Nursing, Zunyi Medical University. My research topic is "Sinicization of independent preference Index and preliminary application in cancer patients". In view of your knowledge and rich clinical experience, we sincerely invite you to be a consulting expert in this study. In order to ensure the rigor and accuracy of the scale, please evaluate the professional theoretical knowledge and clinical work experience, from the content of the items, language expression habits, cultural background and other aspects, evaluate the relevance of the items and the theme, and put forward your valuable suggestions for modification.

This study aims to sinicalize the "autonomous preference index quantity table", which includes two dimensions: medical decision preference and medical information acquisition preference. The scale consists of two parts: the information acquisition scale of 8 general items and the decision scale of 15 items, with a total of 23 items. Your suggestions will be used as an important basis for us to build the scale items, I sincerely hope that you will put forward valuable opinions, please in your busy schedule to give guidance and help, I would like to express my most sincere thanks!

Thank you sincerely for your support and help, and I wish you a smooth filling explanation:

1. The following are the preliminary entries formed after Chinese translation. Please evaluate the correlation of each entry of the scale with "patient autonomous preference" with the following level 1~4 scoring method.1 means "irrelevant", 2 is "weak correlation, 3 is" strong correlation, 4 is " complete correlation, please fill in the score you think appropriate in the corresponding column.

2. If any items are modified or deleted, please fill in the contents and reasons in the column of "modify or delete opinions"; to add the items, please fill in the column of "Supplementary Suggestions", and explain the importance and reasons.

3. Please send your comments to muyanchao0520@163.com within one week. If you have any questions, please call 15822419105. Thank you again for your guidance and help in this topic. I wish you good health and a happy family!

Yan Chao( Zunyi Medical University)

Mentor: Li Yonghong

**Part 1: Chinese version of the independent preference Index**

Note: You choose 1~4 points to evaluate the intelligibility of the entry content and the entry content, the correlation between the language expression habits and cultural background in China. This scale consists of two parts: the information acquisition scale of 8 general items and the decision scale of 15 items. The decision scale includes six general entries and three illustrations related to nine entries.(No correlation =1 point, weak correlation =2 points, strong correlation =3 points, strong correlation =4 points).

**Part 2: Expert situation questionnaire**

Fill in the form: The survey is only used for the overall survey of the consulting experts in this study, and follows the principle of confidentiality. Please fill in the relevant information in the box conforming to your actual situation, and type "" in the selected part. If you need further explanation, please mark after selection.

Table 1 Basic information questionnaire of experts

| Name |  | work unit |  | | |
| --- | --- | --- | --- | --- | --- |
| Age |  | research area |  | Engage in professional years |  |
| professional ranks and titles |  | | | educational background |  |

**Thank you sincerely again for your help and support to this topic!**

**《自主偏好指数》专家咨询表**

尊敬的专家：

您好！感谢您在百忙之中阅读此咨询表！

我是遵义医科大学护理学院研究生晏超，我的研究课题是“自主偏好指数的汉化及在癌症患者中的初步应用”，鉴于您的学识和丰富的临床经验，我们诚邀您为本研究的咨询专家。为了确保量表的严谨性和准确性，请您根据专业理论知识和临床工作经验，**从条目内容的可理解性、语言表达习惯、文化背景等方面逐一评议，对此量表的各条目与主题相关性进行评价，并提出您的宝贵修改意见。**

本研究旨在汉化“自主偏好指数量表”，**该量表包括两个维度：医疗决策偏好和医疗信息获取偏好。**量表由两个部分组成:8项一般条目的信息获取量表及15项条目的决策量表，共23个条目。您的建议将作为我们构建量表条目的重要依据，真诚希望您提出宝贵的意见，敬请您在百忙之中给予指导和帮助，对此谨表示最诚挚的感谢！

衷心感谢您的支持和帮助，祝工作顺利！

**填写说明：**

1.以下是翻译汉化后形成的初步条目，请用以下的 1～4 级评分方法**评价这份量表各个条目与“患者自主偏好”的相关性**。1 分代表“不相关”，2分代表“弱相关，3 分代表“较强相关，4 分代表“完全相关，请将您认为合适的分值填在相应的栏目内。

2.若有修改或删除条目，请在“修改或删除意见”栏内填写修改内容及修改原因；**若要增加条目，请在“补充建议”栏内填写，并说明重要程度及原因。**

[3.恳请您在一周内将意见反馈至muyanchao0520@163.com，如有疑问请致电15822419105。再次感谢您对本课题的指导和帮助，祝您身体健康，阖家辛福！](mailto:3.恳请您在一周内将意见反馈至muyanchao0520@163.com，如有疑问请致电15822419105。再次感谢您对本课题的指导和帮助，祝您身体健康，阖家辛福！)

遵义医科大学研究生：晏超

导师：李永红

第一部分：中文版自主偏好指数

说明：**您选择1～4分来评价条目内容与条目内容的可理解性、我国语言表达习惯、文化背景的相关性，**该量表由两个部分组成：8项一般条目的信息获取量表及15项条目的决策量表。决策量表包括6个一般条目和3个插图相关的9个条目。(无相关=1分，弱相关=2分，较强相关=3分，强相关=4分)。

**第二部分：专家情况调查表**

填表说明：该调查内容仅用于本研究咨询专家的整体情况调查，并遵循保密原则，请您在符合您实际情况的框内填入相关信息，选择部分打“√”，如需进一步说明，请在选择后标注。

表1专家基本情况调查表

| 姓名 |  | 工作单位 |  | | |
| --- | --- | --- | --- | --- | --- |
| 年龄 |  | 研究方向 |  | 从事专业年限 |  |
| 职称 |  | | | 学历 |  |

**再次诚挚地感谢您对本课题的帮助和支持！**
